# Association of Weekend Catch-Up Sleep With the Triglyceride/High-Density Lipoprotein Cholesterol Ratio

**DOI:** 10.7759/cureus.85429

**Published:** 2025-06-05

**Authors:** Moon-Kyung Shin, Dongyeop Kim, Tae-Jin Song

**Affiliations:** 1 Ewha Medical Research Institute, Ewha Womans University College of Medicine, Seoul, KOR; 2 Department of Neurology, Ewha Womans University Seoul Hospital, Ewha Womans University College of Medicine, Seoul, KOR

**Keywords:** dyslipidemia, insulin resistance, sleep deprivation, triglyceride/high-density lipoprotein cholesterol ratio, weekend catch-up sleep

## Abstract

Purpose: Insulin resistance affects more than just diabetes, influencing overall health. Weekend catch-up sleep (WCUS) might alleviate the adverse health outcomes of ongoing sleep deprivation. The triglyceride/high-density lipoprotein cholesterol (TG/HDL) ratio serves as an effective marker for insulin resistance. This study aimed to investigate the association between WCUS and the TG/HDL ratio according to gender and weekday sleep duration.

Methods: We analyzed nationwide data from the 2019-2021 Korean National Health and Nutrition Examination Survey (KNHANES), which included 10,562 participants: 4,611 male participants (43.7%) and 5,951 female participants (56.3%). Participants' sleep data were collected through self-reported questionnaires. WCUS was divided into the following categories: ≤0 hours, >0-1 hours, >1-2 hours, and >2 hours. Weekday sleep was categorized into three groups: ≤6 hours, >6-8 hours, and >8 hours.

Results: The number of participants and their percentages for WCUS were as follows: 7,152 (62.7%) for ≤0 hours, 1,163 (11.6%) for >0-1 hours, 1,275 (14.3%) for >1-2 hours, and 972 (11.5%) for >2 hours. In multivariable analysis, WCUS (>2 hours) was inversely associated with the TG/HDL ratio (ß coefficient = -0.22, 95% confidence interval (CI): -0.38, -0.06, p = 0.006; odds ratio (OR) = 0.74, 95% CI: 0.57, 0.96, p = 0.031 for TG/HDL > 3.9), and with weekday sleep < 6 hours (OR = 0.68, 95% CI: 0.46, 0.99, p = 0.025). Considering gender differences, the association between WCUS (>2 hours) and the TG/HDL ratio showed a borderline inverse correlation, particularly among male participants (β = -0.41, 95% CI: -0.70, -0.12, p = 0.006; OR = 0.69, 95% CI: 0.51, 0.94, p = 0.052 for TG/HDL > 3.9; OR = 0.65, 95% CI: 0.41, 1.03, p = 0.087 for weekday sleep < 6). No significant association between WCUS (>2 hours) and the TG/HDL ratio was observed in female participants.

Conclusion: The WCUS (>2 hours) had an inverse association with the TG/HDL ratio in male participants, which may have a beneficial effect on insulin resistance.

## Introduction

Insulin resistance is a common metabolic disorder closely linked with type 2 diabetes mellitus (DM) [1]. This condition occurs when the body's cells become less responsive to insulin, a hormone essential for regulating blood sugar levels [2]. The implications of insulin resistance extend beyond DM, affecting a wide range of health issues including hypertension, dyslipidemia, liver diseases, cardiovascular diseases, certain types of cancer, obesity, and infectious diseases [2-6]. Moreover, insulin resistance is a key factor in the development of atherogenic dyslipidemia, a condition characterized by elevated levels of triglycerides (TG), low levels of high-density lipoprotein (HDL) cholesterol, and an increase in small low-density lipoprotein (LDL) particles. This lipid profile is particularly atherogenic, meaning it promotes the formation of plaques in the arteries, increasing the risk of cardiovascular diseases [6].

Sleep accounts for one-third of our lives and plays a critical role in overall health, with chronic sleep deprivation posing significant risks to metabolic function and cardiovascular health. In addition, poor sleep quality and sleep disorders, including obstructive sleep apnea, can exacerbate these risks, contributing to insulin resistance and atherogenic dyslipidemia [7-9]. In modern society, habitual sleep restriction during workdays is often unavoidable due to demanding lifestyles and work schedules. To counteract this sleep deprivation, individuals frequently engage in catch-up sleep (CUS) on their days off, typically referred to as weekend CUS (WCUS), by extending sleep duration during free days to compensate for lost sleep on workdays. This pattern of sleep has the potential to positively influence health, particularly with regard to vascular risk factors such as dyslipidemia, hypertension, and insulin resistance [10,11]. Various studies have suggested that this compensatory sleep approach may help mitigate the adverse health outcomes associated with chronic sleep deprivation [10-12].

The TG/HDL ratio serves as a simple and practical surrogate marker for insulin resistance and atherogenic dyslipidemia [13,14]. This index has gained recognition for its ease of use and cost-effectiveness, especially in settings where more direct and complex measurements of insulin resistance are not readily available. The ratio provides a valuable tool for assessing metabolic health in various clinical settings and identifying individuals at risk of complications associated with insulin resistance and atherogenic dyslipidemia [15,16].

Therefore, we aimed to investigate the association between WCUS duration and the TG/HDL ratio, stratified by gender and weekday sleep duration.

## Materials and methods

Study design and participants

The KNHANES is a cross-sectional survey periodically conducted to track the relationship between risk factors, including health and nutritional status, and major chronic diseases in a representative sample of the Korean population aged one year and older. In summary, KNHANES was designed to enroll nationally representative samples using a complex, multistage, stratified, and clustered sampling design based on the Korean National Census Registry. It includes interviews on health behavior and nutrition, as well as health examinations (such as biospecimen sampling and anthropometric measurements). The survey was conducted every three years from 1998 to 2005 and annually since 2007. More detailed information on KNHANES can be found elsewhere [17].

**Figure 1:**
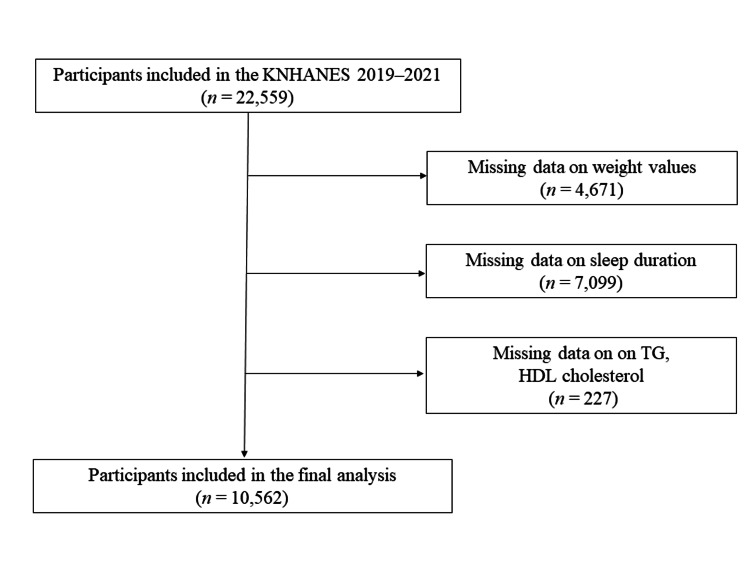
Flowchart of the participant selection process. KNHANES, Korea National Health and Nutrition Examination Survey; TG, triglyceride; HDL cholesterol, high-density lipoprotein cholesterol

Of the 22,559 participants, 4,671 were excluded due to missing weight data, 7,099 due to missing sleep duration information, and 227 due to unavailable TG or HDL cholesterol levels, leaving 10,562 participants remaining (Figure 1). This study adhered to the Helsinki Declaration guidelines for ethical approval and participant consent. Our institutional review board approved the study of Ewha Womans University Seoul Hospital (SEUMC 2024-03-025), with the study duration from March 26, 2024, to February 26, 2025. Since the data are publicly accessible through the official website of KNHANES (https://knhanes.kdca.go.kr), the need for ethical approval and informed consent was waived.

Assessment of weekend catch-up sleep

The sleep questionnaire queried bedtimes and wake-up times for weekdays and weekends, as detailed in Table 9 in the Appendices [18,19]. The weekday sleep duration was calculated from responses to “Bedtime on weekdays” and “Wake-up time on weekdays,” categorized into three groups: ≤6 hours, >6-8 hours, and >8 hours. The weekend sleep duration was derived similarly from weekend responses. Total sleep duration was computed using the formula: (5 × weekday sleep duration + 2 × weekend sleep duration)/7. The duration of WCUS was calculated by subtracting weekday sleep hours from weekend sleep hours, with participants categorized into four WCUS groups: ≤0 hours, >0-1 hour, >1-2 hours, and >2 hours [18-20].

Assessment of the TG/HDL ratio

Blood samples were collected in the morning after at least eight hours of fasting. After proper procedures, including serum separation, samples were immediately refrigerated, transferred to the central laboratory, and analyzed within 24 hours. Total cholesterol, TG, and HDL cholesterol levels were measured using an enzymatic method [17]. The TG/HDL ratio was defined as TG in mg/dL divided by HDL in mg/dL.

Covariates

Previous studies identified potential confounders associated with sleep [19,20]. These included age, sex, body mass index (BMI), household income (low, low middle, middle high, high, or missing/non-respondent), education level (elementary, middle, high school, college and above, or missing/non-respondent), smoking status (never, former, current, or missing/non-respondent), alcohol consumption (two or more times per week, or missing/non-respondent), physical activity (regular aerobic exercise, categorized as yes or no, or missing/non-respondent), comorbidities (defined as the prevalence of diabetes and hypertension, categorized as yes or no, or missing/non-respondent), statin use (defined as taking medication, categorized as yes or no, or missing/non-respondent), and total sleep duration.

Statistical analysis

Analyses were conducted using SAS software (version 9.4) with complex sampling design data. Multistage, stratified, clustered values served as survey weights, ensuring appropriate sample weights for combined survey cycles in all analyses [17]. To check for selection bias due to random exclusion from the total participants, we compared the differences in general characteristics between included (final) and excluded participants using t-tests and Chi-squared tests. Additionally, we compared the differences in clinical characteristics between male participants and female participants using the same statistical methods.

The TG/HDL ratio was divided into quintiles, resulting in five ordinal levels (0, 1, 2, 3, 4). We compared the differences in clinical characteristics stratified by TG/HDL ratio quintiles using a generalized linear model for continuous variables, while the Chi-squared test was used for categorical variables.

We performed univariable and multivariable regression models to examine the association between WCUS duration and the TG/HDL ratio. First, we used linear regression models to evaluate linear associations, treating WCUS as an ordinal variable converted into dummy variables (0, 1), and the TG/HDL ratio as a continuous variable. These models estimated regression coefficients (β) with 95% confidence intervals (CIs). Second, for a more intuitive presentation of the results, we applied logistic regression models, categorizing the TG/HDL ratio into quintiles. The lowest quintile (Q1) was used as the reference group. In this analysis, WCUS was treated as an ordinal variable, and the TG/HDL ratio as quintiles, with odds ratios (ORs) and 95% CIs calculated. Furthermore, we also examined the association between the WCUS duration and the TG/HDL ratio stratified by weekday sleep duration using multivariable linear and logistic regression models. Models were adjusted for covariates. To reduce selection bias, missing covariate values were included in the regression analysis. Using regression imputation, predictions were made based on non-missing variables, and these predicted values were substituted as if they were actual data, allowing for valid treatment in complex sample analysis [19]. To assess normal distribution, an additional model adjusted for the TG/HDL ratio divided into quintiles was also analyzed for the multivariable linear regression models.

## Results

General characteristics compared between included and excluded participants

Table 1 outlines the general characteristics of the participants, divided into those who were included (final participants) and those who were excluded. The included participants tended to be older, female, have lower income levels, higher education levels, and were more likely to smoke, consume alcohol, and be physically active compared to those who were excluded (49.9 vs. 40.8 years, p < .001; 5,951 (56.3%) vs. 6,254 (52.1%), p < .001; 1,978 (18.8%) vs. 1,926 (16.2%), p < .001; 3,556 (35.8%) vs. 2,773 (26.8%), p < .001; 1,584 (15.1%) vs. 1,371 (12.6%), p < .001; 1,948 (18.6%) vs. 1,581 (14.6%), p < .001; 3,944 (42.8%) vs. 3,046 (41.1%), p = 0.024).

**Table 1:** General characteristics compared between included and excluded participants Data are presented as mean ± standard deviation or the number of sample size and its percentage. Independent t-test for continuous variables and Chi-squared test for categorical variables were used.

	n = 22,559		
	Excluded	Included (Final participants)	
	n = 11,997	n = 10,562	p-value
Age, years	40.8 ± 24.6	49.9 ± 19.0	< 0.001
Gender, n (%)			< 0.001
Male	5743 (47.9)	4611 (43.7)	
Female	6254 (52.1)	5951 (56.3)	
Household income, n (%)	n = 11,927	n = 10,526	< 0.001
Low	1926 (16.2)	1978 (18.8)	
Low middle	3038 (25.5)	2642 (25.1)	
Middle high	3436 (28.8)	2823 (26.8)	
High	3527 (29.6)	3083 (29.3)	
Education level, n (%)	n = 10,342	n = 9,933	< 0.001
≤Elementary school	3999 (38.7)	2077 (20.9)	
Middle school	970 (9.4)	1144 (11.5)	
High school	2600 (25.1)	3156 (31.8)	
≥College	2773 (26.8)	3556 (35.8)	
Smoking status, n (%)	n = 10,844	n = 10,465	< 0.001
Never	7558 (69.7)	6665 (63.7)	
Former	1915 (17.7)	2216 (21.2)	
Current	1371 (12.6)	1584 (15.1)	
Drinking status, n (%)	n = 10,853	n = 10,470	< 0.001
< 2 times per week	9272 (85.4)	8522 (81.4)	
≥2 times per week	1581 (14.6)	1948 (18.6)	
Physical activity, n (%)	n = 7,418	n = 9,214	0.024
No	4372 (58.9)	5270 (57.2)	
Yes	3046 (41.1)	3944 (42.8)	

General characteristics according to the study population

Table 2 presents the general characteristics of participants by gender. Male participants were more likely to have a higher BMI, smoke, consume alcohol, and engage in physical activity (24.7 vs. 23.2 kg/m², p < .001; 1,300 (30.0%) vs. 284 (5.5%), p < .001; 1,363 (29.3%) vs. 585 (10.5%), p < .001; 1,863 (49.1%) vs. 2,081 (41.4%), p < .001). However, female participants were more likely to be older and have higher rates of statin use activity (983 (13.5%) vs. 590 (10.4%), p < .001). Sleep questionnaire data indicated that male participants were more likely to have a longer WCUS and weekend sleep duration compared to female participants (0.9 vs. 0.7 hours, p = 0.001; 7.6 vs. 7.5 hours, p = 0.022). Clinical data indicated that male participants were more likely to have higher fasting glucose, TG, and TG/HDL ratio, and a lower HDL level compared to female participants (102.2 vs. 97.5 mg/dL, p < .001; 150.9 vs. 107.7 mg/dL, p < .001; 3.5 vs. 2.2, p < .001; 48.3 vs. 56.1 mg/dL, p < .001).

**Table 2:** General characteristics according to the study population by gender TG, triglyceride; HDL, high-density lipoprotein; TG/HDL, triglyceride/high-density lipoprotein cholesterol ratio; h, hours. Data are presented as mean ± standard error or the number of sample size and its percentage. The percentages used in this table reflect weighted percentages derived from data obtained through a complex sampling design. Independent t-test for continuous variables and chi-squared test for categorical variables were used.

	Total	Male participants	Female participants	
	n = 10,562	n = 4,611	n = 5,951	p-value
Age, years	45.8 ± 0.3	44.8 ± 0.4	46.7 ± 0.4	< 0.001
Body mass index, kg/m^2^	23.9 ± 0.1	24.7 ± 0.1	23.2 ± 0.1	< 0.001
Household income, n (%)	n = 10,562	n = 4,597	n = 5,929	< 0.001
Low	1978 (14.5)	775 (12.8)	1203 (16.1)	
Low middle	2642 (23.6)	1140 (23.2)	1502 (24.1)	
Middle high	2823 (28.6)	1269 (29.4)	1554 (27.9)	
High	3083 (33.3)	1413 (34.6)	1670 (31.9)	
Education level, n (%)	n = 9,933	n = 4,343	n = 5,590	< 0.001
≤Elementary school	2077 (14.7)	720 (11.1)	1357 (18.3)	
Middle school	1144 (10.1)	508 (9.9)	636 (10.2)	
High school	3156 (34.6)	1472 (36.6)	1684 (32.6)	
≥College	3556 (40.6)	1643 (42.4)	1913 (38.8)	
Smoking status, n (%)	n = 10,465	n = 4,573	n = 5,892	< 0.001
Never	6665 (60.2)	1440 (32.8)	5225 (87.6)	
Former	2216 (22.1)	1833 (37.3)	383 (6.9)	
Current	1584 (17.8)	1300 (30.0)	284 (5.5)	
Drinking status, n (%)	n = 10,470	n = 4,575	n = 5,895	< 0.001
< 2 times per week	8522 (80.1)	3212 (70.7)	5310 (89.5)	
≥2 times per week	1948 (19.9)	1363 (29.3)	585 (10.5)	
Physical activity, n (%)	n = 9,214	n = 3,956	n = 5,258	< 0.001
No	5270 (54.8)	2093 (50.9)	3177 (58.6)	
Yes	3944 (45.2)	1863 (49.1)	2081 (41.4)	
Diabetes, n (%)	n = 10,561	n = 4,611	n = 5,950	0.068
No	9537 (92.4)	4129 (91.9)	5408 (92.9)	
Yes	1024 (7.6)	482 (8.1)	542 (7.1)	
Hypertension, n (%)	n = 10,562	n = 4,611	n = 5,951	0.186
No	8133 (82.1)	3546 (82.0)	4587 (82.3)	
Yes	2429 (17.9)	1065 (18.0)	1364 (17.7)	
Statin use, n (%)	n = 10,561	n = 4,610	n = 5,951	< 0.001
No	8988 (88.0)	4020 (89.6)	4968 (86.5)	
Yes	1573 (12.0)	590(10.4)	983 (13.5)	
Total sleep duration, h	7.0 ± 0.0	7.0 ± 0.0	7.0 ± 0.0	0.529
Weekend catch-up sleep duration, h	0.8 ± 0.0	0.9 ± 0.0	0.7 ± 0.0	0.001
Weekday sleep duration, h	6.8 ± 0.0	6.8 ± 0.0	6.8 ± 0.0	0.689
Weekend sleep duration, h	7.6 ± 0.0	7.6 ± 0.0	7.5 ± 0.0	0.022
Fasting glucose, mg/dL	99.9 ± 0.3	102.2 ± 0.4	97.5 ± 0.3	< 0.001
TG, mg/dL	129.3 ± 1.4	150.9 ± 2.3	107.7 ± 1.1	< 0.001
HDL cholesterol, mg/dL	52.2 ± 0.2	48.3 ± 0.2	56.1 ± 0.2	< 0.001
TG/HDL ratio	2.8 ± 0.0	3.5 ± 0.1	2.2 ± 0.0	< 0.001

Clinical characteristics according to the TG/HDL ratio quintile

**Figure 2:**
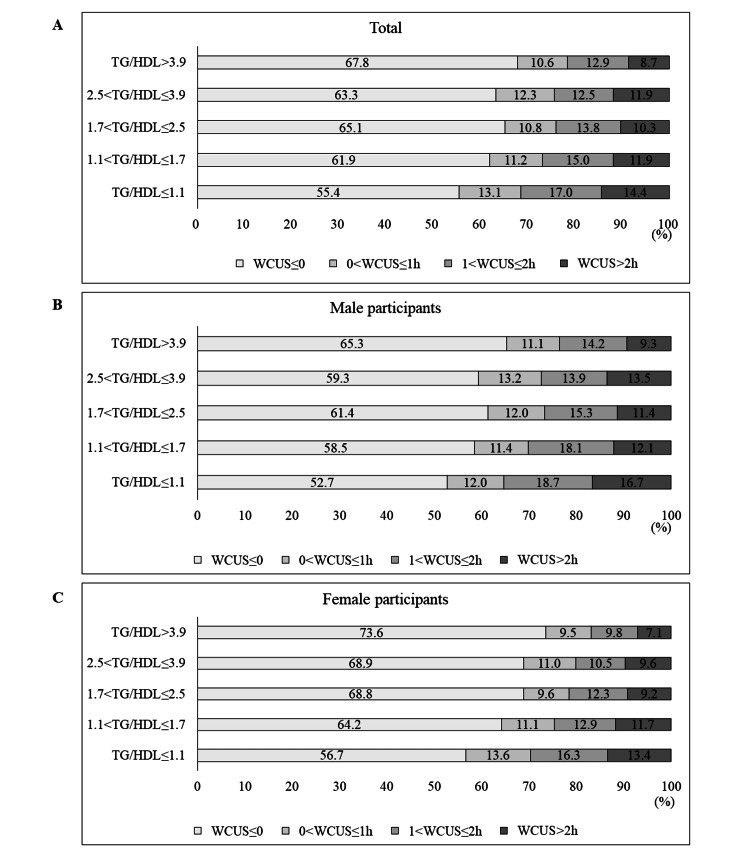
Frequency of the weekend catch-up sleep duration according to the triglyceride/high-density lipoprotein cholesterol ratio by quintile A. Frequency of weekend catch-up sleep duration according to the triglyceride/high-density lipoprotein cholesterol ratio by quintile in all participants. B. Frequency of the weekend catch-up sleep duration according to the triglyceride/high-density lipoprotein cholesterol ratio by quintile in male participants. C. Frequency of the weekend catch-up sleep duration according to the triglyceride/high-density lipoprotein cholesterol ratio by quintile in female participants. WCUS, weekend catch-up sleep; TG/HDL, triglyceride/high-density lipoprotein cholesterol; h, hours.

Table 3 and Figure 2 illustrate the frequency of WCUS duration according to the TG/HDL ratio quintile by gender, showing that the highest TG/HDL quintiles are more likely to have a lower rate of WCUS duration longer than two hours compared to the lowest TG/HDL quintiles in both male participants and female participants (84 (16.7%) vs. 100 (9.3%), p = 0.001 for male participants; 177 (13.4%) vs. 43 (7.1%), p < .001 for female participants).

**Table 3:** Frequency of the weekend catch-up sleep duration according to the triglyceride/high-density lipoprotein cholesterol ratio quintile TG/HDL, triglyceride/high-density lipoprotein cholesterol ratio; WCUS, weekend catch-up sleep; h, hours. Data are presented as the number of sample size and its percentage. The percentages used in this table reflect weighted percentages derived from data obtained through a complex sampling design.

	Total	TG/HDL					
		Quintile 1	Quintile 2	Quintile 3	Quintile 4	Quintile 5	
	n (%)	n (%)	n (%)	n (%)	n (%)	n (%)	p-value
		TG/HDL≤1.1	1.1	1.7	2.5	TG/HDL>3.9	< 0.001
Total		2113 (20.9)	2110 (19.2)	2114 (19.8)	2113 (19.7)	2112 (20.4)	
≤0 h	7152 (62.7)	1262 (55.4)	1409 (61.9)	1477 (65.1)	1465 (63.3)	1539 (67.8)	
>0–1 h	1163 (11.6)	268 (13.1)	249 (11.2)	210 (10.8)	240 (12.3)	196 (10.6)	
>1–2 h	1275 (14.3)	322 (17.0)	255 (15.0)	245 (13.8)	219 (12.5)	234 (12.9)	
>2 h	972 (11.5)	261 (14.4)	197 (11.9)	182 (10.3)	189 (11.9)	143 (8.7)	
		TG/HDL≤1.1	1.1	1.7	2.5	TG/HDL>3.9	0.001
Male		592 (12.9)	736 (15.6)	908 (19.6)	1075 (23.1)	1300 (28.8)	
≤0 h	3024 (60.5)	339 (52.7)	474 (58.5)	604 (61.4)	705 (59.3)	902 (65.3)	
>0–1 h	523 (11.9)	73 (12.0)	87 (11.4)	102 (12.0)	130 (13.2)	131 (11.1)	
>1–2 h	604 (15.5)	96 (18.7)	100 (18.1)	111 (15.3)	130 (13.9)	167 (14.2)	
>2 h	460 (12.1)	84 (16.7)	75 (12.1)	91 (11.4)	110 (13.5)	100 (9.3)	
		TG/HDL≤1.1	1.1	1.7	2.5	TG/HDL>3.9	< 0.001
Female		1521 (28.9)	1374 (22.8)	1206 (20.0)	1038 (16.3)	812 (12.0)	
≤0 h	4128 (64.9)	923 (56.7)	935 (64.2)	873 (68.8)	760 (68.9)	637 (73.6)	
>0–1 h	640 (11.3)	195 (13.6)	162 (11.1)	108 (9.6)	110 (11.0)	65 (9.5)	
>1–2 h	671 (13.0)	226 (16.3)	155 (12.9)	134 (12.3)	89 (10.5)	67 (9.8)	
>2 h	512 (10.8)	177 (13.4)	122 (11.7)	91 (9.2)	79 (9.6)	43 (7.1)	

Table 4 presents the clinical characteristics categorized by TG/HDL ratio quintile and gender. The quintiles were ≤1.1, >1.1-1.7, >1.7-2.5, >2.5-3.9, and >3.9. In both male participants and female participants, higher TG/HDL quintiles were associated with significant increases in age, BMI, prevalence of diabetes and hypertension, statin use, increased fasting glucose, and TG levels, along with decreased HDL cholesterol (47.9 vs. 37.7 years, p < .001 for male participants; 54.8 vs. 40.4 years, p < .001 for female participants; 26.2 vs. 22.4 kg/m^2^, p < .001 for male participants; 25.5 vs. 21.4 kg/m^2^, p < .001 for female participants; 173 (10.9%) vs. 34 (3.8%), p < .001 for male participants; 143 (15.0%) vs. 57 (2.9%), p < .001 for female participants; 340 (21.3%) vs. 84 (10.4%), p < .001 for male participants; 282 (30.9%) vs. 167 (8.1%), p < .001 for female participants; 183 (11.1%) vs. 39 (5.5%), p < .001 for male participants; 161 (19.1%) vs. 154 (7.9%), p < .001 for female participants; 109.7 vs. 93.8 mg/dL, p < .001 for male participants; 109.1 vs. 91.3 mg/dL, p < .001 for female participants; 277.6 vs. 52.2 mg/dL, p < .001 for male participants; 234.7 vs. 54.5 mg/dL, p < .001 for female participants; 39.7 vs. 61.5 mg/dL, p < .001 for male participants; 41.5 vs. 66.7 mg/dL, p < .001 for female participants). In male participants, the highest TG/HDL quintiles were more likely to have a shorter total sleep duration compared to the lowest TG/HDL quintiles (6.9 vs. 7.2 hours, p = 0.007).

**Table 4:** Clinical characteristics according to the triglyceride/high-density lipoprotein cholesterol ratio quintile IQR, interquartile range; TG/HDL, triglyceride/high-density lipoprotein cholesterol ratio; TG, triglyceride; HDL, high-density lipoprotein; h, hours. Data are presented as mean ± standard error or the number of sample size and its percentage. The percentages used in this table reflect weighted percentages derived from data obtained through a complex sampling design. Generalized linear models for continuous variables and chi-squared test for categorical variables were used.

	Total			Male participants			Female participants		
	Quintile 1	Quintile 5		Quintile 1	Quintile 5		Quintile 1	Quintile 5	
	TG/HDL≤1.1	TG/HDL>3.9	p-value	TG/HDL≤1.1	TG/HDL>3.9	p-value	TG/HDL≤1.1	TG/HDL>3.9	p-value
	n = 2113	n = 2112		n = 592	n = 1300		n = 1521	n = 812	
TG/HDL, mean	0.8 ± 0.0	6.8 ± 0.1		0.9 ± 0.0	7.3 ± 0.2		0.8 ± 0.0	5.8 ± 0.1	
TG/HDL, median	0.9 ± 0.0	5.4 ± 0.1		0.9 ± 0.0	5.7 ± 0.1		0.8 ± 0.0	5.0 ± 0.1	
TG/HDL, IQR	0.7 - 1.0	4.4 - 7.4		0.7 - 1.0	4.5 - 7.9		0.7 - 1.0	4.3 - 6.4	
Age, years	39.5 ± 0.4	50.0 ± 0.4	< 0.001	37.7 ± 0.8	47.9 ± 0.5	< 0.001	40.4 ± 0.5	54.8 ± 0.8	< 0.001
Body mass index, kg/m^2^	21.7 ± 0.1	26.0 ± 0.1	< 0.001	22.4 ± 0.1	26.2 ± 0.1	< 0.001	21.4 ± 0.1	25.5 ± 0.2	< 0.001
Household income, n (%)	n = 2103	n = 2107	< 0.001	n = 589	n = 1296	0.969	n = 1514	n = 811	< 0.001
Low	279 (10.6)	426 (15.4)		100 (12.6)	203 (11.8)		179 (9.7)	223 (24.1)	
Low middle	510 (23.3)	548 (24.5)		149 (25.1)	319 (23.5)		361 (22.5)	229 (26.8)	
Middle high	598 (29.1)	552 (28.0)		161 (28.0)	365 (29.7)		437 (29.6)	187 (23.9)	
High	716 (37.0)	581 (32.1)		179 (34.3)	409 (35.0)		537 (38.3)	172 (25.1)	
Education level, n (%)	n = 2020	n = 1938	< 0.001	n = 558	n = 1214	< 0.001	n = 1462	n = 724	< 0.001
≤Elementary school	306 (10.8)	435 (15.9)		118 (13.1)	167 (9.7)		188 (9.7)	268 (31.2)	
Middle school	200 (9.1)	209 (9.1)		68 (11.0)	125 (8.2)		132 (8.3)	84 (11.0)	
High school	667 (35.0)	597 (33.3)		208 (42.3)	399 (34.0)		459 (31.7)	198 (31.4)	
≥College	847 (45.2)	697 (41.8)		164 (33.6)	523 (48.0)		683 (50.3)	174 (26.4)	
Smoking status, n (%)	n = 2098	n = 2086	< 0.001	n = 587	n = 1288	< 0.001	n = 1511	n = 798	0.018
Never	1646 (76.5)	978 (41.8)		303 (52.0)	275 (22.5)		1343 (87.6)	703 (88.1)	
Former	292 (14.8)	565 (28.0)		184 (29.9)	523 (37.8)		108 (8.1)	42 (4.2)	
Current	160 (8.6)	543 (30.3)		100 (18.1)	490 (39.6)		60 (4.4)	53 (7.7)	
Drinking status, n (%)	n = 2099	n = 2088	< 0.001	n = 587	n = 1289	< 0.001	n = 1512	n = 799	0.001
< 2 times per week	1757 (83.8)	1594 (74.1)		446 (77.7)	850 (66.5)		1311 (86.6)	744 (92.3)	
≥2 times per week	342 (16.2)	494 (25.9)		141 (22.3)	439 (33.5)		201 (13.4)	55 (7.7)	
Physical activity, n (%)	n = 1799	n = 1881	< 0.001	n = 440	n = 1182	< 0.001	n = 1359	n = 699	< 0.001
No	934 (49.3)	1147 (59.0)		203 (44.1)	679 (56.3)		731 (51.4)	468 (65.8)	
Yes	865 (50.7)	734 (41.0)		237 (55.9)	503 (43.7)		628 (48.6)	231 (34.2)	
Diabetes, n (%)	n = 2113	n = 2112	< 0.001	n = 592	n = 1300	< 0.001	n = 1521	n = 812	< 0.001
No	2022 (96.8)	1796 (87.9)		558 (96.2)	1127 (89.1)		1464 (97.1)	669 (85.0)	
Yes	91 (3.2)	316 (12.1)		34 (3.8)	173 (10.9)		57 (2.9)	143 (15.0)	
Hypertension, n (%)	n = 2113	n = 2112	< 0.001	n = 592	n = 1300	< 0.001	n = 1521	n = 812	< 0.001
No	1862 (91.2)	1490 (75.9)		508 (89.6)	960 (78.7)		1354 (91.9)	530 (69.1)	
Yes	251 (8.8)	622 (24.1)		84 (10.4)	340 (21.3)		167 (8.1)	282 (30.9)	
Statin use, n (%)	n = 2112	n = 2112	< 0.001	n = 591	n = 1300	< 0.001	n = 1521	n = 812	< 0.001
No	1919 (92.9)	1768 (86.6)		552 (94.5)	1117 (88.9)		1367 (92.1)	651 (80.9)	
Yes	193 (7.1)	344 (13.4)		39 (5.5)	183 (11.1)		154 (7.9)	161 (19.1)	
Total sleep duration, h	7.1 ± 0.0	6.9 ± 0.0	0.008	7.2 ± 0.1	6.9 ± 0.0	0.007	7.0 ± 0.0	7.0 ± 0.1	0.100
Weekend catch-up sleep, h	1.0 ± 0.0	0.6 ± 0.0	< 0.001	1.1 ± 0.1	0.7 ± 0.0	< 0.001	0.9 ± 0.0	0.5 ± 0.1	< 0.001
Weekday sleep duration, h	6.8 ± 0.0	6.7 ± 0.0	0.353	6.9 ± 0.1	6.7 ± 0.0	0.247	6.8 ± 0.0	6.8 ± 0.1	0.512
Weekend sleep duration, h	7.8 ± 0.0	7.4 ± 0.0	< 0.001	8.0 ± 0.1	7.4 ± 0.1	< 0.001	7.7 ± 0.1	7.3 ± 0.1	< 0.001
Fasting glucose, mg/dL	92.1 ± 0.3	109.5 ± 0.8	< 0.001	93.8 ± 0.7	109.7 ± 1.0	< 0.001	91.3 ± 0.3	109.1 ± 1.2	< 0.001
TG, mg/dL	53.8 ± 0.3	265.0 ± 3.7	< 0.001	52.2 ± 0.6	277.6 ± 5.0	< 0.001	54.5 ± 0.3	234.7 ± 3.2	< 0.001
HDL cholesterol, mg/dL	65.1 ± 0.3	40.2 ± 0.2	< 0.001	61.5 ± 0.6	39.7 ± 0.2	< 0.001	66.7 ± 0.4	41.5 ± 0.3	< 0.001

In both male participants and female participants, the highest TG/HDL quintiles were more likely to have a shorter WCUS and weekend sleep duration compared to the lowest TG/HDL quintiles (0.7 vs. 1.1 hours, p < .001 for males; 0.5 vs. 0.9 hours, p < .001 for females; 7.4 vs. 8.0 hours, p < .001 for male participants; 7.3 vs. 7.7 hours, p < .001 for female participants).

Associations between the WCUS duration and the TG/HDL ratio

Table 5 shows the linear regression analyses for the associations between the WCUS duration and the TG/HDL ratio. A WCUS lasting >2 hours was associated with a significantly inverse TG/HDL ratio compared to ≤0 hours (β = -0.22, 95% confidence interval (CI): -0.38 to -0.06, p = 0.006). Similarly, in male participants, a WCUS lasting >2 hours was associated with a significantly inverse TG/HDL ratio compared to ≤0 hours (β = -0.41, 95% CI: -0.70 to -0.12, p = 0.006), while female participants showed no significant association.

**Table 5:** Linear regression analysis for association between the weekend catch-up sleep duration and the triglyceride/high-density lipoprotein cholesterol ratio ß coefficients; CI, confidence interval; TG/HDL, triglyceride/high-density lipoprotein cholesterol ratio; WCUS, weekend catch-up sleep; h, hours. ^a^Adjusted for age, sex, body mass index, household income, education, smoking, drinking, physical activity, diabetes, hypertension, statin use, total sleep duration, TG/HDL ratio into quintiles.

	TG/HDL					
	Total		Male		Female	
	ß (95% CI)	p-value	ß (95% CI)	p-value	ß (95% CI)	p-value
Age adjusted						
≤0 h	Ref.		Ref.		Ref.	
>0–1 h	0.00 (-0.21, 0.22)	0.983	-0.01 (-0.38, 0.37)	0.970	-0.04 (-0.21, 0.12)	0.608
>1–2 h	-0.02 (-0.26, 0.22)	0.883	-0.16 (-0.57, 0.24)	0.421	0.01 (-0.16, 0.18)	0.918
>2 h	-0.16 (-0.37, 0.05)	0.126	-0.37 (-0.71, -0.03)	0.031	0.01 (-0.13, 0.16)	0.871
p trend	0.040		0.072		0.900	
Multivariable adjusted^a^						
≤0 h	Ref.		Ref.		Ref.	
>0–1 h	-0.03 (-0.19, 0.13)	0.694	-0.14 (-0.44, 0.17)	0.369	0.02 (-0.09, 0.13)	0.697
>1–2 h	-0.05 (-0.23, 0.13)	0.564	-0.14 (-0.47, 0.20)	0.420	0.05 (-0.06, 0.16)	0.369
>2 h	-0.22 (-0.38, -0.06)	0.006	-0.41 (-0.70, -0.12)	0.006	-0.03 (-0.11, 0.05)	0.521
p trend	0.046		0.032		0.837	

Table 6 shows the logistic regression analyses for associations between the WCUS duration and the TG/HDL ratio quintile. Compared to WCUS ≤ 0 hours, a WCUS lasting >2 hours was significantly inversely associated with the highest quintile of the TG/HDL ratio (OR = 0.74, 95% CI: 0.57-0.96, p = 0.031 for TG/HDL > 3.9). In male participants, although not statistically significant, a WCUS lasting >2 hours showed a borderline inverse association with the highest quintile, compared to a WCUS of ≤ 0 hours (OR = 0.69, 95% CI: 0.51-0.94, p = 0.052 for TG/HDL > 3.9). However, in female participants, a WCUS lasting >2 hours showed no significant association.

**Table 6:** Logistic regression analysis for association between weekend catch-up sleep duration and the triglyceride/high-density lipoprotein cholesterol ratio OR, odds ratio; CI, confidence interval; TG/HDL, triglyceride/high-density lipoprotein cholesterol ratio; WCUS, weekend catch-up sleep; h, hours; No. of participants, number of participants. ^a^Adjusted for age, sex, body mass index, household income, education, smoking, drinking, physical activity, diabetes, hypertension, statin use, total sleep duration

	TG/HDL									
	Quintile 1		Quintile 2		Quintile 3		Quintile 4		Quintile 5	
	OR (95% CI)	p-value	OR (95% CI)	p-value	OR (95% CI)	p-value	OR (95% CI)	p-value	OR (95% CI)	p-value
Age adjusted										
Total	TG/HDL≤1.1		1.1		1.7		2.5		TG/HDL>3.9	
No. of participants	2113		2110		2114		2113		2112	
≤0 h	1.00		1.00		1.00		1.00		1.00	
>0–1 h	1.04 (0.87, 1.24)	0.780	0.86 (0.72, 1.03)	0.397	0.92 (0.77, 1.11)	0.617	1.25 (1.04, 1.51)	0.264	0.98 (0.81, 1.18)	0.901
>1–2 h	1.03 (0.86, 1.22)	0.915	0.92 (0.77, 1.10)	0.881	0.99 (0.83, 1.19)	0.646	1.05 (0.86, 1.28)	0.194	1.03 (0.85, 1.24)	0.407
>2 h	1.01 (0.84, 1.22)	0.920	0.88 (0.72, 1.06)	0.538	0.93 (0.76, 1.13)	0.646	1.36 (1.09, 1.69)	0.041	0.88 (0.70, 1.10)	0.238
p trend	0.971		0.300		0.727		0.016		0.650	
Male	TG/HDL≤1.1		1.1		1.7		2.5		TG/HDL>3.9	
No. of participants	592		736		908		1075		1300	
≤0 h	1.00		1.00		1.00		1.00		1.00	
>0–1 h	0.87 (0.63, 1.20)	0.269	0.84 (0.62, 1.14)	0.397	1.01 (0.79, 1.29)	0.813	1.34 (1.04, 1.72)	0.197	0.95 (0.75, 1.21)	0.726
>1–2 h	1.00 (0.74, 1.35)	0.966	1.03 (0.78, 1.36)	0.304	0.99 (0.75, 1.30)	0.970	1.05 (0.80, 1.36)	0.215	0.96 (0.76, 1.22)	0.622
>2 h	1.12 (0.83, 1.52)	0.270	0.84 (0.62, 1.13)	0.370	0.94 (0.70, 1.26)	0.682	1.42 (1.07, 1.91)	0.083	0.79 (0.59, 1.05)	0.137
p trend	0.633		0.483		0.977		0.044		0.459	
Female	TG/HDL≤1.1		1.1		1.7		2.5		TG/HDL>3.9	
No. of participants	1521		1374		1206		1038		812	
≤0 h	1.00		1.00		1.00		1.00		1.00	
>0–1 h	1.20 (0.98, 1.48)	0.170	0.90 (0.71, 1.13)	0.757	0.84 (0.62, 1.13)	0.337	1.12 (0.84, 1.48)	0.788	0.97 (0.68, 1.39)	0.872
>1–2 h	1.14 (0.91, 1.42)	0.457	0.87 (0.69, 1.09)	0.493	1.00 (0.79, 1.27)	0.518	0.99 (0.74, 1.32)	0.409	1.00 (0.70, 1.41)	0.994
>2 h	0.96 (0.75, 1.24)	0.235	0.93 (0.72, 1.20)	0.950	0.93 (0.70, 1.23)	0.899	1.24 (0.88, 1.75)	0.279	1.01 (0.69, 1.47)	0.909
p trend	0.190		0.543		0.689		0.533		0.999	
Multivariable adjusted										
Total	TG/HDL≤1.1		1.1		1.7		2.5		TG/HDL>3.9	
No. of participants	2113		2110		2114		2113		2112	
≤0 h	1.00		1.00		1.00		1.00		1.00	
>0–1 h	1.04 (0.86, 1.26)	0.384	0.84 (0.68, 1.03)	0.283	0.86 (0.70, 1.05)	0.283	1.33 (1.08, 1.62)	0.064	0.99 (0.79, 1.24)	0.317
>1–2 h	1.19 (0.97, 1.45)	0.379	0.96 (0.79, 1.16)	0.574	1.00 (0.82, 1.21)	0.465	0.99 (0.81, 1.21)	0.070	0.93 (0.75, 1.15)	0.825
>2 h	1.24 (0.98, 1.58)	0.216	0.88 (0.69, 1.11)	0.620	0.91 (0.74, 1.13)	0.734	1.31 (1.04, 1.66)	0.133	0.74 (0.57, 0.96)	0.031
p trend	0.201		0.320		0.411		0.011		0.151	
Male	TG/HDL≤1.1		1.1		1.7		2.5		TG/HDL>3.9	
No. of participants	592		736		908		1075		1300	
≤0 h	1.00		1.00		1.00		1.00		1.00	
>0–1 h	0.94 (0.63, 1.41)	0.201	0.85 (0.59, 1.21)	0.584	0.93 (0.70, 1.25)	0.852	1.43 (1.10, 1.86)	0.125	0.90 (0.69, 1.17)	0.607
>1–2 h	1.30 (0.91, 1.86)	0.320	1.05 (0.76, 1.44)	0.256	0.98 (0.73, 1.32)	0.818	1.04 (0.79, 1.37)	0.137	0.87 (0.67, 1.13)	0.879
>2 h	1.38 (0.91, 2.10)	0.193	0.78 (0.53, 1.13)	0.249	0.91 (0.67, 1.22)	0.675	1.52 (1.12, 2.07)	0.066	0.69 (0.51, 0.94)	0.052
p trend	0.263		0.466		0.895		0.010		0.120	
Female	TG/HDL≤1.1		1.1		1.7		2.5		TG/HDL>3.9	
No. of participants	1521		1374		1206		1038		812	
≤0 h	1.00		1.00		1.00		1.00		1.00	
>0–1 h	1.11 (0.89, 1.38)	0.934	0.85 (0.66, 1.09)	0.446	0.79 (0.58, 1.09)	0.207	1.20 (0.88, 1.64)	0.234	1.14 (0.76, 1.70)	0.438
>1–2 h	1.13 (0.88, 1.45)	0.761	0.88 (0.69, 1.11)	0.608	1.01 (0.78, 1.32)	0.426	0.89 (0.65, 1.21)	0.227	1.03 (0.71, 1.51)	0.849
>2 h	1.17 (0.88, 1.55)	0.559	0.96 (0.71, 1.28)	0.717	0.93 (0.68, 1.28)	0.993	1.03 (0.70, 1.53)	0.948	0.87 (0.55, 1.39)	0.399
p trend	0.539		0.458		0.536		0.550		0.808	

Association between the WCUS duration and the TG/HDL ratio stratified by the weekday sleep duration

Table 7 shows the association between the WCUS duration and the TG/HDL ratio, stratified by the weekday sleep duration, as presented in linear regression analyses. Those with a WCUS lasting >2 hours in the subgroup with a weekday sleep duration of less than six hours were inversely associated with the TG/HDL ratio compared to those with a WCUS ≤0 hours (β = -0.25, 95% CI: -0.52 to 0.02, p = 0.070). In male participants, those with a WCUS lasting >2 hours in the subgroup with a weekday sleep duration of less than six hours were inversely associated with the TG/HDL ratio compared to those with a WCUS ≤0 hours (β = -0.42, 95% CI: -0.89 to 0.06, p = 0.085), while no association was found in female participants.

**Table 7:** Linear regression analysis for association between the weekend catch-up sleep duration and the triglyceride/high-density lipoprotein cholesterol ratio, stratified by the weekday sleep duration ß coefficients; CI, confidence interval; TG/HDL, triglyceride/high-density lipoprotein cholesterol ratio; WCUS, weekend catch-up sleep; h, hours. Adjusted for age, sex, body mass index, household income, education, smoking, drinking, physical activity, diabetes, hypertension, statin use, total sleep duration, TG/HDL ratio into quintiles.

	TG/HDL					
	Total		Male		Female	
	ß (95% CI)	p-value	ß (95% CI)	p-value	ß (95% CI)	p-value
≤6 h						
≤0	Ref.		Ref.		Ref.	
0 - 1h	-0.06 (-0.31, 0.19)	0.634	-0.36 (-0.79, 0.07)	0.100	0.16 (-0.07, 0.40)	0.176
1 - 2h	0.04 (-0.27, 0.36)	0.785	0.00 (-0.57, 0.56)	0.987	0.09 (-0.10, 0.29)	0.353
>2h	-0.25 (-0.52, 0.02)	0.070	-0.42 (-0.89, 0.06)	0.085	-0.06 (-0.21, 0.08)	0.405
p trend	0.262		0.226		0.960	
>6–8						
≤0	Ref.		Ref.		Ref.	
0 - 1h	-0.01 (-0.24, 0.21)	0.915	0.04 (-0.40, 0.47)	0.870	-0.06 (-0.16, 0.04)	0.214
1 - 2h	-0.14 (-0.34, 0.06)	0.177	-0.32 (-0.67, 0.02)	0.067	0.04 (-0.11, 0.18)	0.594
>2h	-0.14 (-0.35, 0.07)	0.198	-0.38 (-0.80, 0.04)	0.073	0.05 (-0.09, 0.19)	0.494
p trend	0.139		0.049		0.614	
>8 h						
≤0	Ref.		Ref.		Ref.	
0 - 1h	-0.22 (-0.68, 0.24)	0.343	-1.07 (-2.13, -0.01)	0.047	-0.13 (-0.44, 0.17)	0.386
1 - 2h	-0.44 (-0.83, -0.04)	0.030	-0.19 (-0.99, 0.61)	0.636	-0.37 (-0.78, 0.05)	0.084
>2h	0.04 (-0.56, 0.65)	0.891	0.34 (-0.81, 1.49)	0.563	0.10 (-0.87, 1.07)	0.838
p trend	0.203		0.958		0.347	

Table 8 shows the association between the WCUS duration and the TG/HDL ratio quintile, stratified by the weekday sleep duration, as presented in logistic regression analyses. In the subgroup with a weekday sleep duration of less than six hours, those with a WCUS lasting >2 hours were significantly inversely associated with the highest quintile of the TG/HDL ratio compared to those with WCUS ≤ 0 hours (OR = 0.68, 95% CI: 0.46-0.99, p = 0.025 for TG/HDL > 3.9). In male participants, although not statistically significant, a WCUS lasting >2 hours in the subgroup with a weekday sleep duration of less than six hours showed a borderline inverse association with the highest quintile of the TG/HDL ratio compared to a WCUS of ≤ 0 hours (OR = 0.65, 95% CI: 0.41-1.03, p = 0.087 for TG/HDL > 3.9). However, in female participants, a WCUS lasting >2 hours showed no significant association with the weekday sleep duration subgroups.

**Table 8:** Logistic regression analysis for association between the weekend catch-up sleep duration and the triglyceride/high-density lipoprotein cholesterol ratio, stratified by the weekday sleep duration OR, odds ratio; CI, confidence interval; TG/HDL, triglyceride/high-density lipoprotein cholesterol ratio; WCUS, weekend catch-up sleep; h, hours; NA, no association; No. of participants, number of participants. Adjusted for age, sex, body mass index, household income, education, smoking, drinking, physical activity, diabetes, hypertension, statin use, total sleep duration.

	TG/HDL									
	Quintile 1		Quintile 2		Quintile 3		Quintile 4		Quintile 5	
	OR (95% CI)	p-value	OR (95% CI)	p-value	OR (95% CI)	p-value	OR (95% CI)	p-value	OR (95% CI)	p-value
Total	TG/HDL≤1.1		1.1		1.7		2.5		TG/HDL>3.9	
≤6 h										
No. of participants	871		855		888		928		910	
≤0 h	1.00		1.00		1.00		1.00		1.00	
>0–1 h	1.14 (0.86, 1.51)	0.482	0.79 (0.57, 1.09)	0.470	0.77 (0.54, 1.09)	0.151	1.31 (0.95, 1.82)	0.120	1.08 (0.76, 1.55)	0.193
>1–2 h	1.41 (1.06, 1.88)	0.140	0.88 (0.66, 1.18)	0.804	0.98 (0.73, 1.32)	0.664	0.92 (0.70, 1.21)	0.087	0.93 (0.68, 1.28)	0.824
>2 h	1.40 (0.97, 2.03)	0.318	0.79 (0.55, 1.13)	0.486	1.01 (0.72, 1.42)	0.508	1.19 (0.85, 1.65)	0.498	0.68 (0.46, 0.99)	0.025
p trend	0.086		0.388		0.520		0.205		0.163	
>6–8										
No. of participants	1020		1009		985		954		916	
≤0 h	1.00		1.00		1.00		1.00		1.00	
>0–1 h	1.01 (0.77, 1.33)	0.368	0.88 (0.67, 1.15)	0.350	0.92 (0.72, 1.18)	0.940	1.30 (0.99, 1.70)	0.196	0.94 (0.71, 1.26)	0.941
>1–2 h	1.17 (0.86, 1.59)	0.720	1.05 (0.79, 1.41)	0.460	0.98 (0.69, 1.38)	0.581	0.97 (0.69, 1.35)	0.260	0.91 (0.64, 1.28)	0.826
>2 h	1.33 (0.91, 1.93)	0.213	0.96 (0.64, 1.44)	0.941	0.77 (0.50, 1.19)	0.285	1.28 (0.85, 1.90)	0.404	0.89 (0.57, 1.39)	0.776
p trend	0.445		0.742		0.602		0.218		0.915	
>8 h										
No. of participants	222		246		241		231		286	
≤0 h	1.00		1.00		1.00		1.00		1.00	
>0–1 h	0.79 (0.24, 2.58)	0.895	1.00 (0.29, 3.44)	0.807	1.88 (0.54, 6.52)	0.841	0.88 (0.13, 5.92)	0.911	0.10 (0.01, 0.81)	0.131
>1–2 h	0.63 (0.18, 2.17)	0.770	0.45 (0.13, 1.54)	0.176	3.22 (0.92, 11.21)	0.391	0.89 (0.16, 4.94)	0.892	0.68 (0.14, 3.40)	0.368
>2 h	0.60 (0.10, 3.65)	0.765	1.32 (0.30, 5.85)	0.480	3.19 (0.55, 18.41)	0.530	0.55 (0.05, 5.66)	0.666	0.29 (0.02, 3.47)	0.793
p trend	0.801		0.564		0.185		0.968		0.143	
Male	TG/HDL≤1.1		1.1		1.7		2.5		TG/HDL>3.9	
≤6 h										
No. of participants	231		300		376		461		565	
≤0 h	1.00		1.00		1.00		1.00		1.00	
>0–1 h	1.04 (0.53, 2.05)	0.305	0.89 (0.52, 1.55)	0.974	0.64 (0.37, 1.13)	0.224	1.80 (1.17, 2.75)	0.028	0.91 (0.57, 1.45)	0.671
>1–2 h	1.83 (1.06, 3.15)	0.076	1.00 (0.62, 1.61)	0.510	0.83 (0.52, 1.32)	0.997	1.07 (0.72, 1.60)	0.183	0.86 (0.58, 1.28)	0.890
>2 h	1.67 (0.86, 3.23)	0.322	0.74 (0.41, 1.31)	0.284	0.90 (0.56, 1.44)	0.662	1.47 (0.95, 2.26)	0.403	0.65 (0.41, 1.03)	0.087
p trend	0.130		0.689		0.447		0.039		0.326	
>6–8										
No. of participants	286		355		440		500		587	
≤0 h	1.00		1.00		1.00		1.00		1.00	
>0–1 h	0.98 (0.58, 1.65)	0.856	0.81 (0.51, 1.28)	0.424	1.18 (0.83, 1.68)	0.297	1.17 (0.80, 1.72)	0.806	0.89 (0.63, 1.26)	0.921
>1–2 h	0.97 (0.55, 1.70)	0.828	1.25 (0.74, 2.11)	0.153	1.01 (0.63, 1.63)	0.964	1.10 (0.71, 1.71)	0.537	0.86 (0.57, 1.29)	0.896
>2 h	1.13 (0.58, 2.18)	0.672	0.75 (0.33, 1.70)	0.464	0.85 (0.44, 1.62)	0.466	1.73 (1.00, 2.99)	0.099	0.77 (0.42, 1.40)	0.564
p trend	0.980		0.477		0.734		0.241		0.728	
>8 h										
No. of participants	75		81		92		114		148	
≤0 h	1.00		1.00		1.00		1.00		1.00	
>0–1 h	NA		2.10 (0.13, 34.25)	0.465	3.00 (0.25, 36.76)	0.861	2.33 (0.04, 137.69)	0.013	NA	
>1–2 h	0.75 (0.06, 9.54)	0.005	0.21 (0.01, 4.41)	0.216	12.26 (1.61, 93.29)	0.038	NA		NA	
>2 h	0.68 (0.02, 28.40)	0.044	1.25 (0.10, 16.35)	0.702	1.10 (0.05, 24.53)	0.463	0.56 (0.03, 10.85)	0.039	NA	
p trend			0.657		0.086					
Female	TG/HDL≤1.1		1.1		1.7		2.5		TG/HDL>3.9	
≤6 h										
No. of participants	640		555		512		467		345	
≤0 h	1.00		1.00		1.00		1.00		1.00	
>0–1 h	1.27 (0.90, 1.78)	0.661	0.73 (0.48, 1.10)	0.414	0.94 (0.58, 1.51)	0.464	0.80 (0.50, 1.28)	0.764	1.44 (0.86, 2.40)	0.092
>1–2 h	1.24 (0.85, 1.79)	0.796	0.78 (0.53, 1.15)	0.681	1.18 (0.78, 1.77)	0.545	0.75 (0.48, 1.17)	0.442	1.14 (0.68, 1.92)	0.610
>2 h	1.30 (0.85, 1.98)	0.571	0.83 (0.53, 1.29)	0.995	1.20 (0.72, 1.99)	0.517	0.87 (0.53, 1.42)	0.905	0.72 (0.35, 1.45)	0.138
p trend	0.381		0.357		0.796		0.514		0.282	
>6–8										
No. of participants	734		654		545		454		329	
≤0 h	1.00		1.00		1.00		1.00		1.00	
>0–1 h	1.02 (0.73, 1.41)	0.251	0.94 (0.67, 1.31)	0.791	0.70 (0.47, 1.02)	0.294	1.49 (1.04, 2.14)	0.009	1.04 (0.60, 1.82)	0.967
>1–2 h	1.33 (0.92, 1.93)	0.360	0.92 (0.65, 1.29)	0.668	0.92 (0.59, 1.45)	0.510	0.75 (0.46, 1.24)	0.171	0.95 (0.51, 1.77)	0.722
>2 h	1.43 (0.92, 2.23)	0.243	1.03 (0.65, 1.64)	0.718	0.73 (0.41, 1.30)	0.541	0.84 (0.46, 1.53)	0.486	1.14 (0.61, 2.16)	0.669
p trend	0.266		0.926		0.248		0.068		0.967	
>8 h										
No. of participants	147		165		149		117		138	
≤0 h	1.00		1.00		1.00		1.00		1.00	
>0–1 h	0.79 (0.24, 2.65)	0.971	0.82 (0.20, 3.40)	0.640	1.92 (0.47, 7.87)	0.819	0.63 (0.12, 3.32)	0.646	0.10 (0.01, 1.24)	0.216
>1–2 h	0.67 (0.12, 3.85)	0.863	0.57 (0.13, 2.41)	0.916	1.46 (0.27, 7.90)	0.511	2.32 (0.36, 14.97)	0.198	0.60 (0.05, 6.96)	0.633
>2 h	0.67 (0.03, 15.10)	0.906	0.29 (0.03, 2.98)	0.414	8.63 (0.78, 95.97)	0.123	0.39 (0.02, 6.98)	0.461	0.33 (0.01, 10.52)	0.919
p trend	0.939		0.662		0.308		0.626		0.328	

Subgroup analysis of the WCUS duration and TG/HDL ratio by covariates

**Figure 3:**
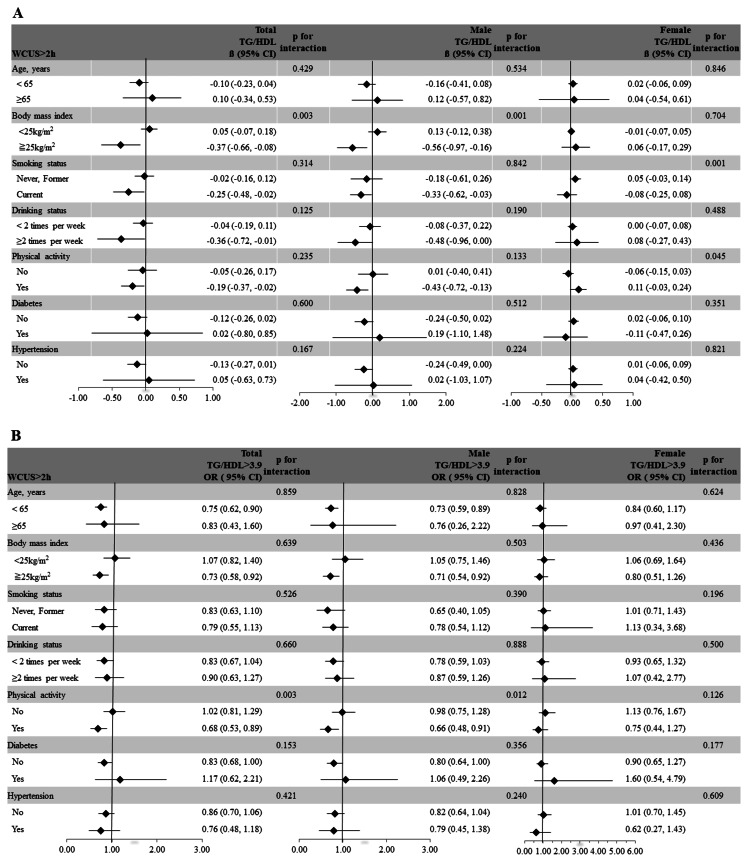
Subgroup analysis of the weekend catch-up sleep duration and triglyceride/high-density lipoprotein cholesterol ratio by covariates A. Subgroup analysis of the weekend catch-up sleep duration and triglyceride/high-density lipoprotein cholesterol ratio by covariates in linear regression analyses. B. Subgroup analysis of the weekend catch-up sleep duration and triglyceride/high-density lipoprotein cholesterol ratio by covariates in logistic regression analyses. ß, regression coefficient; OR, odds ratio; CI, confidence interval; WCUS, weekend catch-up sleep; h, hours; TG/HDL, triglyceride/high-density lipoprotein cholesterol.

Figure 3 illustrates the associations between the WCUS duration and the TG/HDL ratio by subgroups in linear and logistic regression analyses. Figure 3A shows that in linear regression analyses, a significant inverse association was found between WCUS and the TG/HDL ratio among participants with a BMI ≥ 25 kg/m², current smokers, heavy drinkers, and physically active individuals. In male participants, these results were consistent, with significant inverse associations observed in those with a BMI ≥ 25 kg/m², current smokers, heavy drinkers, and physically active individuals, while no association was found in female participants. Notably, significant interaction effects were noted only for BMI, particularly in subgroups with a BMI ≥ 25 kg/m² (β = -0.37, 95% CI: -0.66, -0.08, p for interaction = 0.003 for all participants; β = -0.56, 95% CI: -0.97, -0.16, p for interaction = 0.001 for male participants).

Figure 3B shows that in logistic regression analyses, a significant inverse association was found between WCUS and the TG/HDL ratio among participants younger than 65 years, those with a BMI ≥ 25 kg/m², physically active individuals, and those with no history of diabetes. In male participants, these results were consistent, with significant inverse associations observed in those younger than 65 years, with a BMI ≥ 25 kg/m², physically active, and with no history of diabetes, while no association was found in females. Notably, significant interaction effects were noted only for physical activity, particularly in subgroups of physically active individuals (OR = 0.68, 95% CI: 0.53, 0.89, p for interaction = 0.003 for all participants; OR = 0.66, 95% CI: 0.48, 0.91, p for interaction = 0.012 for male participants).

## Discussion

The key findings of our study include an inverse association between the WCUS duration and the TG/HDL ratio. Longer WCUS durations were associated with lower TG/HDL ratios, suggesting a beneficial effect on metabolic health. To examine whether the weekday sleep duration influences the metabolic benefits of WCUS, further stratified analysis was conducted based on the weekday sleep duration. Weekday sleep durations of six hours or less, combined with WCUS lasting >2 hours, had a significant inverse association with the TG/HDL ratio. Our findings specifically show that when WCUS lasts more than two hours, there is a significant inverse association with the TG/HDL ratio only for those who sleep less than six hours on weekdays. Conversely, for individuals who get sufficient sleep on weekdays (6-8 hours or more than eight hours), WCUS lasting over two hours does not show a significant inverse association with the TG/HDL ratio. This suggests that WCUS may vary depending on differences in sleep duration and that the duration of sleep deprivation might affect WCUS. These findings suggest that individuals with shorter weekday sleep may need a longer WCUS duration to achieve metabolic benefits. This association was significant in male participants, while no significant association was found in female participants, indicating a potential sex-specific influence on metabolic health. Typically, there are gender differences in how epidemiological factors affect metabolic health. In male participants, smoking, alcohol consumption, and physical inactivity are associated with a higher risk of metabolic disease. In female participants, low socioeconomic status, poor diet, and lack of physical activity are associated with a higher risk of metabolic disease [21,22]. Therefore, the impact of these epidemiological factors may differ between genders, with varying thresholds for influencing metabolic health. Further research is needed to better identify and understand these gender-specific associations.

The TG/HDL ratio is linked to various health issues, including metabolic syndrome, cerebrovascular disease, coronary artery disease, and peripheral arterial disease, indicating its importance in cardiovascular and metabolic health [23]. The TG/HDL ratio may serve as a marker for insulin resistance and atherosclerosis risk, and elevated levels have been linked to severe illness and higher mortality in COVID-19 patients [24]. Moreover, a previous study showed a significant association between the TG/HDL ratio and long-term all-cause mortality in patients with coronary artery disease, underscoring its prognostic value in cardiovascular health [25]. Our study provides insights into the association between the TG/HDL ratio and WCUS. Previous research in South Korea has shown significant associations between WCUS and various metabolic risk factors, including obesity, prediabetes, dyslipidemia, and metabolic syndrome [19,20,26,27]. This suggests that WCUS may help reduce metabolic risks and counteract the negative effects of insufficient weekday sleep.

WCUS may help partially reverse the adverse metabolic effects of chronic sleep deprivation. While no studies have specifically examined its impact on the TG/HDL ratio, research on metabolic markers like insulin sensitivity following CUS offers insights into potential effects of WCUS on metabolic health. In a randomized crossover study conducted in Australia [28] with 19 men experiencing chronic weekday sleep restriction followed by WCUS, insulin sensitivity improved by up to 45% after three consecutive weekend nights of 10-hour sleep extension compared to continuous sleep restriction of six hours per night. However, another study [29] conducted in the United States using a randomized experimental design reported conflicting results, indicating that WCUS did not restore whole-body or tissue-specific insulin sensitivity. In that study, participants in the WCUS group experienced a five-hour sleep restriction during the workweek and only gained about 1.1 hours of additional sleep over the weekend. This modest increase may have been inadequate to observe significant protective effects, indicating that longer or more consistent recovery sleep might be essential for metabolic benefits. This notion is further supported by a crossover study conducted in Thailand [30] with 21 participants experiencing chronic sleep deprivation, defined as sleeping ≤ 6 hours per night. Participants were instructed to maintain or extend sleep duration for two weeks. While overall metabolic parameters remained unchanged with sleep extension, those extending sleep to longer than six hours experienced improvements in homeostatic model assessment of insulin resistance, insulin secretion, and β-cell function, underscoring the importance of adequate sleep for metabolic benefits.

The significance of our findings is reinforced by validation across independent, population-based, cross-sectional studies, highlighting the public health implications of WCUS patterns on metabolic health. However, our study has notable limitations. First, although the data are nationwide and represent a sample from South Korea, there are significant demographic differences between the included (final participants) and excluded participants, suggesting potential selection bias. Consequently, it would be prudent to interpret the generalizability with caution. Second, being retrospective and cross-sectional, the study cannot establish causality between WCUS and the TG/HDL ratio. While we used the TG/HDL ratio as a biomarker, we lacked direct assessments of insulin resistance and atherosclerosis presence or progression, with unclear implications for these conditions. This underscores the need for longitudinal studies to explore causal links between sleep patterns and cardiovascular health, incorporating direct measures of insulin resistance and atherosclerosis. Third, total sleep duration and WCUS were self-reported, which could introduce bias and affect accuracy. Discrepancies between subjective reports and objectively measured sleep may result in differences between actual and reported sleep durations. Fourth, potential confounding factors were not adjusted for, such as sleep disorders like obstructive sleep apnea, the use of hypnotics, and mental health conditions like stress, which could potentially distort our findings.

## Conclusions

Our study shows a significant inverse association between WCUS and the TG/HDL ratio particularly in male participants, indicating a beneficial effect on insulin resistance. However, the beneficial effects for individuals with short weekday sleep (<6 hours) show a significant inverse association with the TG/HDL ratio, which is not observed in those who get sufficient sleep on weekdays (6-8 hours or more). Therefore, WCUS may vary with differences in the weekday sleep duration, and the extent of sleep deprivation could impact WCUS. Nonetheless, the cross-sectional design limits the determination of causality, necessitating further longitudinal research to clarify the relationship between sleep extension and metabolic outcomes.

## Appendices

**Table 9:** Sleep questionnaire details

BP16_1 - Sleep hours - weekdays or workdays														
Code or Value	Value Description													
□□ hours	Range of Values													
88,99	Missing													

BP16_2 - Sleep hours - weekends				
Code or Value	Value Description			
□□ hours	Range of Values			
88,99	Missing			

BP16_11 - Usual bed sleep time on weekdays or workdays (o'clock)				
Code or Value	Value Description			
□□ o'clock	Value was recorded			
88,99	Missing			

BP16_12 - Usual bed sleep time on weekdays or workdays (minutes)				
Code or Value	Value Description			
□□ minutes	Value was recorded			
88,99	Missing			

BP16_13 - Usual wake time on weekdays or workdays (o'clock)				
Code or Value	Value Description			
□□ o'clock	Value was recorded			
88,99	Missing			

BP16_14 - Usual wake time on weekdays or workdays (minutes)				
Code or Value	Value Description			
□□ minutes	Value was recorded			
88,99	Missing			

BP16_21 - Usual bed sleep time on weekends (o'clock)				
Code or Value	Value Description			
□□ o'clock	Value was recorded			
88,99	Missing			

BP16_22 - Usual bed sleep time on weekends (minutes)				
Code or Value	Value Description			
□□ minutes	Value was recorded			
88,99	Missing			

BP16_23 - Usual wake time on weekends (o'clock)				
Code or Value	Value Description			
□□ o'clock	Value was recorded			
88,99	Missing			

BP16_24 - Usual wake time on weekends (minutes)				
Code or Value	Value Description			
□□ minutes	Value was recorded			
88,99	Missing			
